# Unravelling distinct proteome changes in two maize inbreds with contrasting stalk‐lodging resistance upon drought

**DOI:** 10.3389/fpls.2026.1830066

**Published:** 2026-05-05

**Authors:** Rocío C. Arce, Albert Muñoz-Blanxer, Camila Sánchez-Retuerta, Nuria Mauri, David Caparrós-Ruiz

**Affiliations:** Centre for Research in Agricultural Genomics (CRAG, Consorci Consejo Superior de Investigaciones Científicas (CSIC)‐Institut de Recerca i Tecnologia Agroalimentaris (IRTA)‐Universitat Autònoma de Barcelona (UAB)‐Universitat de Barcelona (UB)). Edifici CRAG, Campus de Bellaterra de la Universitat Autònoma de Barcelona (UAB), Cerdanyola del Valles, Barcelona, Spain

**Keywords:** B73, drought, EA2024, proteomics, *Zea mays*

## Abstract

Stalk-lodging and drought are major constraints limiting maize productivity. We previously characterized B73 as stalk-lodging resistant but drought-sensitive maize inbred, while EA2024 is stalk-lodging susceptible and drought-tolerant. Using these inbreds, we performed a comparative proteomic analysis under well-watered and drought conditions. Our results revealed that drought induces extensive proteomic reprogramming in B73, while the EA2024 proteome remains unaltered. Comparative analyses identified 481 differentially accumulated proteins between inbreds across conditions. Among them, 100 proteins were differentially accumulated in well-watered conditions but showed no significant (or attenuated) differences upon drought. We defined these B73 proteins as having a convergent response to drought. Their functional characterization revealed that one third relates to upstream elements of the photosynthetic machinery, suggesting that B73 reprograms its energetic machinery to achieve an organization similar to EA2024. We also identified 381 proteins differentially accumulated between inbreds upon drought, defined as having a divergent response to drought. In this case, B73 over-accumulates proteins mainly involved in cofactors, vitamins metabolism and signaling and under-accumulates proteins linked to downstream photosynthetic components and biosynthetic processes. Thus, the convergent response suggests a partial shift of drought sensitive B73 toward the baseline of drought resistant EA2024. However, the broader divergent response of B73 suggests an inbred-specific metabolic reprogramming associated with reduced energy production, metabolic impairment and altered signaling cascades. Our findings open the possibility that stalk−lodging resistance in B73 was acquired at the expense of drought tolerance, prompting further investigation into the correlation between these two traits across a broader range of maize inbreds.

## 1 Introduction

Maize *(Zea mays* L.) is one of the world most important crops, serving as a primary source of food, feed and biofuel production. However, maize production is constantly challenged by abiotic stresses that occur throughout its life cycle. Among them, drought represents one of the most severe and widespread limitations, substantially reducing growth and yield potential (Sah et al., 2020; Sheoran et al., 2022). In response to drought, plants trigger hormonal signaling and osmotic adjustment. This results in stomatal closure and decreases photosynthetic enzyme production, limiting CO_2_ diffusion into the leaf and consequently limiting carbon assimilation (Tang et al., 2025). The strategy adopted by plants to face these effects determines their degree of tolerance to drought. In drought sensitive plants, the reduction in photosynthetic enzyme production and carbon assimilation causes major shutdown of downstream metabolic processes. Among them, a decrease in ribosomal biosynthesis genes and protein elongation factors (Yadav et al., 2024), and in general dry matter accumulation have been reported in drought sensitive plants (Song et al., 2022). The imbalance between energy production and utilization promotes electron leakage toward alternative acceptors, primarily molecular oxygen, resulting in the formation of reactive oxygen species (ROS). While excessive ROS accumulation can cause oxidative damage to lipids, proteins, and nucleic acids, these molecules also function as key signaling intermediates that activate stress-responsive pathways (Turkan, 2018). Therefore, drought sensitive plants induce antioxidant defense systems to restore redox homeostasis (Dvořák et al., 2021). Together, these coordinated physiological and molecular adjustments aim to minimize oxidative damage, maintain cellular homeostasis, and optimize resource use under limited water availability. On the other hand, drought tolerant plants adapt to this stress without triggering such metabolic changes, maintaining, and even increasing, their level of photosynthetic activity, thus ensuring an adequate energy flux towards the metabolic machinery (Li et al., 2021; Rehaman et al., 2025). Consequently, as the energy utilization of drought tolerant plants mainly remains unaltered, ROS are not over-accumulated, therefore minimal changes are required to readapt their metabolism to drought conditions (Yang et al., 2015).

Drought stress also affects the mechanical properties of maize, as it can weaken stalk strength, increasing the risk of lodging, a major cause of harvest losses (Wang et al., 2023). The plant cell wall must adapt to this new scenario by acting as a dynamic structure that responds to changes in water availability. In a previous study, we characterized a collection of maize inbreds based on the stalk‐lodging resistance, revealing that two of them, B73 and EA2024, had a contrasting behavior; being B73 a lodging‐resistant inbred and EA2024, a lodging-susceptible one (Manga-Robles et al., 2021). We then examined the impact of drought on these two maize inbreds and found that the lodging-resistant B73 is sensitive to this stress, while the lodging-susceptible EA2024 is tolerant (Calderone et al., 2024). Although these two inbreds have a similar photochemical activity in well irrigated conditions, B73 maintains higher levels than EA2024 upon drought. We also determined that B73 displayed a deeper rearrangement of cell walls and induced more substantial changes in gene expression upon drought than EA2024. Similar gene expression alterations have been also characterized in other studies (Liu et al., 2020; Wang et al., 2022). While transcriptomic analyses provide valuable insights into regulatory responses, changes in gene expression do not always translate directly into functional outcomes mainly due to post-translational regulations (Buccitelli and Selbach, 2020). Proteomic approaches have provided further insights into the functional impact of drought on cellular metabolism (Ren et al., 2022). Comparative proteomic analyses between contrasting maize genotypes have shown that drought tolerance is associated with genotype-specific adjustments in photosynthetic machinery and redox homeostasis (Li et al., 2021). In this work, we performed a comparative proteomic analysis of the two contrasting stalk-lodging B73 and EA2024 inbreds grown under control and drought conditions. This approach aimed to identify differentially accumulated proteins (DAPs) and elucidate the molecular mechanisms that could explain their contrasting drought responses, and the relationship between this stress and their stalk-loading resistance. The results provide novel insights into the metabolic and regulatory processes affected by drought, highlighting distinct patterns of proteome responses that shape the adaptive strategies of these two maize inbreds.

## 2 Materials and methods

### 2.1 Plant material and growth conditions

Maize plants were grown in controlled chambers under a 16/8-hour light/dark cycle at 25/22 °C and 50% relative humidity. Once the third leaf was fully expanded, approximately 15 days after sowing, drought treatment was applied by withholding irrigation for 10 days until samples collection. In parallel, control plants were watered regularly and harvested alongside the treated plants. The 10-day drought treatment was selected based on previous experiments (Calderone et al., 2024), in which different durations of water deprivation (7–10 days) were tested and shown to induce a robust drought response.

### 2.2 Protein extraction and digestion

Approximately 1g of leaf tissue from three biological samples of each maize inbred (under control and drought conditions) was ground with liquid nitrogen until a fine powder was obtained. Each powdered sample was then mixed with 10 mL of Tris-Mg/NP-40 extraction buffer (0.5 M Tris–HCl, pH 8.3, 20 mM MgCl_2_ and 2% NP-40) and vortexed vigorously. RuBisCO protein depletion was performed following the protocol described by Kim et al. (2013). Finally, samples were stored at -20 °C in 80% (v/v) acetone.

Samples were sent in cold acetone to the Proteomics Platform of the Parc Científic de Barcelona (Universitat de Barcelona, Spain) for further processing. Briefly, 20 μg of protein from each sample was reduced with 21 mM dithiothreitol (DTT) in 50 mM 500 μL 8 M urea/50 mM NH_4_HCO_3_ (ABC) for 2h at 32°C. Samples were then alkylated using 35mM of Iodoacetamide (IAA) in 50 mM ABC and incubated in the dark for 30 min at room temperature (RT). Afterwards, samples were diluted up to 1 M Urea with 50 mM ABC. Digestion was done in two steps: an initial digestion with 1:20 (w/w) Trypsin 0.2 μg/μL (Sequence grade modified Trypsin, Promega) for 2h at 32°C followed by a digestion with 1:20 (w/w) Trypsin 0.2 μg/μL for 16h at 32°C. Peptides were dried in the Speed Vacuum (Eppendorf) and passed through C18 chromatography (P200 top tip, PolyLC Inc.). Briefly, peptides with 2 μL of 100% formic acid (FA) were charged in the tip columns (previously washed with 70% ACN in 0.1% FA and equilibrated with 0.1% FA) by centrifugation (350 g for 2 min). Columns were washed twice with 100 μL 0.1% FA by centrifugation (350 g for 2 min) and then peptides were eluted in 2 x 80 μL of 70% ACN/0.1% FA by centrifugation (350 g for 2 min and 900 g for 1 min). The peptides were dried in Speed Vacuum (Eppendorf) and stored at -20°C until LC-MS analysis. Our extraction protocol is optimized for soluble proteins and likely underrepresents proteins strongly bound to the membrane or the cell wall.

### 2.3 LC-MS/MS analysis

The dried peptides were resuspended in 2% ACN/1% FA solution, and 600 ng were injected into a nanoAcquity liquid chromatographer coupled to an LTQ-Orbitrap Velos mass spectrometer (Thermo Scientific, Waltham, USA). Peptides were separated on a C18 reverse-phase column with a gradient elution over 175 minutes. Data-dependent acquisition was performed, with peptide masses (m/z 300–1700) analyzed at a resolving power of 60,000. The 15 most abundant peptides per MS scan were fragmented via Collision Induced Dissociation. Raw data was processed using Thermo Xcalibur software (v.2.2).

### 2.4 Mass spectroscopy data analysis

The raw data generated was analyzed with MaxQuant software (v 1.6.3.4) (Cox and Mann, 2008) using standardized workflows. The spectra were searched against the UniProt Proteome *Zea mays* database (v 190121) including contaminants. For Label-free quantification (Cox et al., 2014), the minimum ratio count was set to 2 and both razor and unique peptides were used for quantitation. The False discovery rate (FDR) was set to 1% for both protein and peptide spectrum match levels. Further downstream data processing and statistical tests were carried out with the Perseus software (v 1.6.15.0) using standardized workflows (Tyanova et al., 2016) and all data is described in **Supplementary Table 1**. To minimize the impact of presence-absence variation among maize inbred lines, our comparative analyses were restricted to proteins detected in both EA2024 and B73. Nevertheless, indirect effects of presence-absence variation, such as differences in regulatory components or gene copy number, may still contribute to quantitative differences in protein abundance. Pairwise comparisons were performed with a permutation-based FDR threshold of 0.05. The differentially accumulated proteins with log_2_ fold-change (FC) ≥ 1 at a *p*-value < 0.05 were considered to be significantly over-accumulated proteins, while proteins with log_2_FC ≤ -1 and *p*-value < 0.05 were determined to be significantly under-accumulated. To quantify if each comparison contained biologically meaningful changes, we constructed 2 x 2 contingency tables contrasting the observed number of differentially accumulated proteins and unchanged proteins with a no-change null model. Meaningful changes were then assessed using a two-tailed Fisher’s exact test (Fisher, 1935), and FDR was calculated using Benjamini-Hochberg adjustment (Benjamini and Hochberg, 1995).

### 2.5 Graphical analysis

Principal Component Analyses, fold change distribution curves, volcano plots and enrichment analysis were generated in R language (http://www.r-project.org/) using the ggplot2 package (Wickham, 2009). The heatmap was generated using the pheatmap package (Kolde, 2025), all within the R environment.

## 3 Results

### 3.1 Drought affects the B73 maize proteome, while EA2024 remains largely unaltered

**Figure 1:**
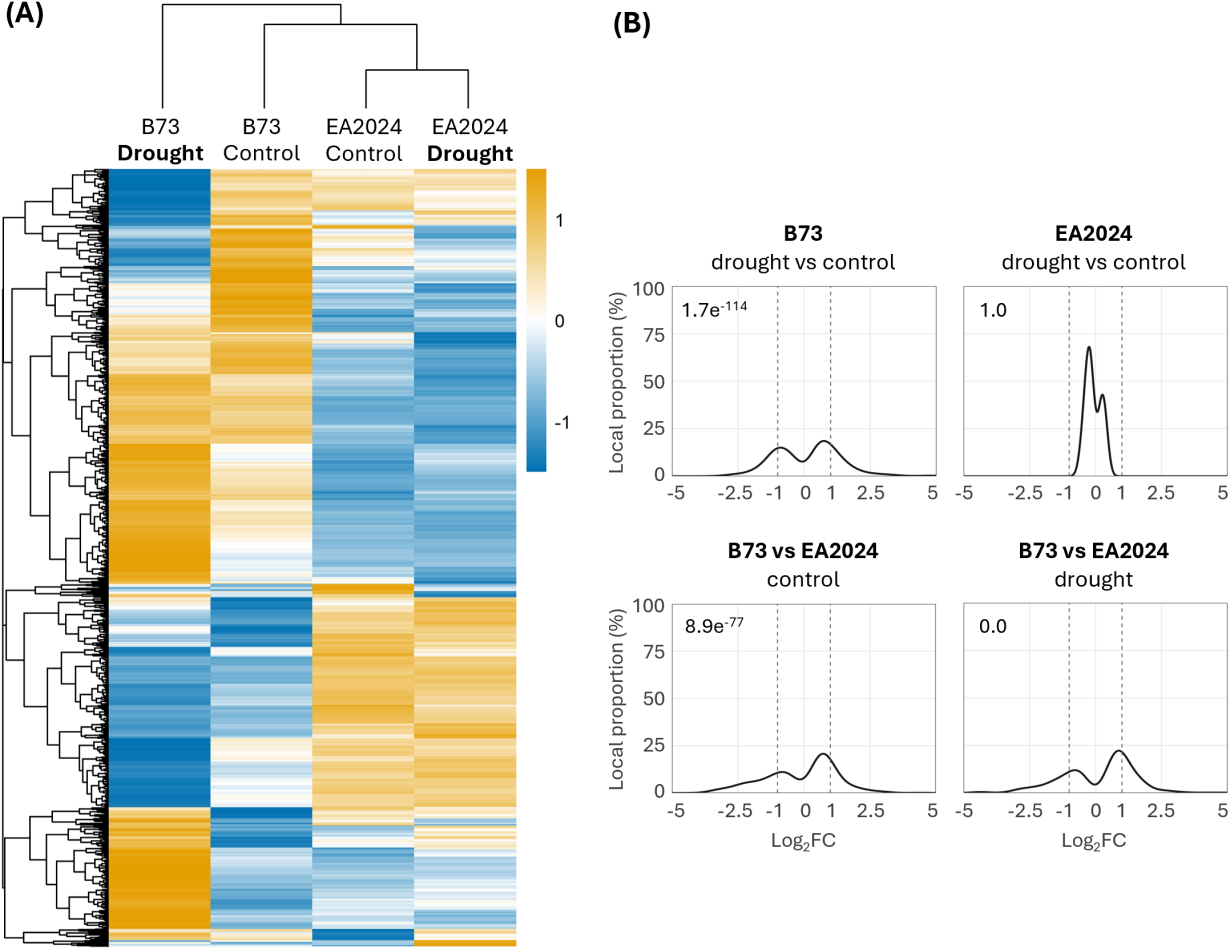
Differential proteomic response of B73 and EA2024 maize inbreds under control and drought conditions. **(A)** hierarchical clustering of protein abundance in the four experimental conditions: B73 under control and drought, and EA2024 under control and drought. The color scale (z-score) represents the extent of over- (orange) or under-accumulation (blue) relative to the mean abundance. **(B)** fold change distribution curves showing the distribution in percentage (local proportion (%), y-axis) of significantly affected proteins (p-value < 0.05) according to Log_2_ fold change (Log_2_FC, x-axis) for drought versus control conditions in each maize inbred (upper left: B73; upper right: EA2024) and for B73 versus EA2024 in control conditions (bottom left) and under drought (bottom right). Quantitative data is provided in **Supplementary Table 1**. Benjamini-Hochberg adjustment of Fisher’s p-values for the assessment of meaningful protein changes in each comparison are shown within each graph, on the top left.

To assess the impact of drought stress on the proteome of B73 and EA2024 inbred lines, we compared protein accumulation profiles of these two inbreds under control and after 10 days of drought. We identified 1674 proteins (**Figure 1A**) and confirmed a normal data distribution of each biological replicate (**Supplementary Figure 1**, **Supplementary Table 1**). The Principal Component Analyses (PCA) of all these proteins revealed a separation between B73 and EA2024 genotypes along the first principal component (PC1), explaining 44.8% of the total variance (**Supplementary Figure 2**). Interestingly, B73 samples also have a distinct shift between control and drought conditions by the second principal component (PC2, 20%), while EA2024 plants cluster together regardless of treatment. In fact, the proteome profile (*z*-score heatmap) of B73 is altered upon drought with respect to control (well-watered) conditions, while the one of EA2024 remains largely unchanged (**Figure 1A**). This indicates that B73 exhibits a higher proteomic response to drought compared to EA2024. To further explore these responses, we generated fold changes distribution curves to compare the proteome of both inbreds in control and in drought conditions (**Figure 1B**). We considered that proteins were differentially abundant when they had a log_2_ FC ≥ 1 or log_2_ FC ≤ -1 at a *p*-value < 0.05, and meaningful differences in each comparison were tested using Fisher’s exact test. Our data revealed that drought affects the proteome of B73 but EA2024 remains unchanged. In addition, our data also revealed significant proteomic differences between inbreds both in control and drought comparisons.

### 3.2 The proteome differences between B73 and EA2024 exhibit convergent and divergent responses to drought

**Figure 2:**
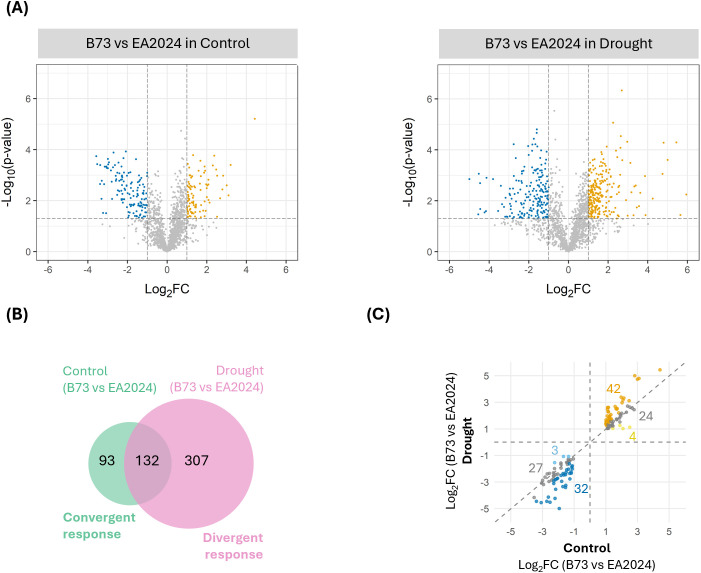
Comparative proteomic responses between B73 and EA2024 maize inbreds under control and drought conditions. **(A)** Volcano plots showing differentially accumulated proteins (DAPs) between B73 and EA2024 maize inbreds under control (left) and drought (right) conditions. Orange and blue dots represent over- and- under-accumulated proteins, respectively (log_2_FC ≥ 1 or Log_2_FC ≤ -1 at *p*-value < 0.05). Gray dots correspond to non-significant changes. **(B)** Venn diagram indicates the specific and shared DAPs identified under control (green) and drought (pink) conditions. Proteins differentially accumulated in control conditions in B73 but with similar abundance in B73 and EA2024 under drought are defined as a convergent response, and proteins with similar abundance in B73 and EA2024 in control conditions but differentially accumulated in B73 under drought are defined as a divergent response. **(C)** Scatter plot is presented containing 132 DAPs in both control and drought conditions (comparing B73 versus EA2024). Orange and dark blue dots represent proteins for which the difference in abundance in drought conditions has increased compared to control conditions (42 and 32 proteins). Yellow and light blue dots represent proteins for which the difference in abundance in drought conditions has decreased compared to control conditions (4 and 3 proteins). Gray dots represent proteins for which the difference in abundance in drought conditions has not changed compared to control conditions (a change of ≤ 20%) (24 and 27 proteins).

To identify the proteins that are differentially abundant between these two contrasting stalk-lodging resistance inbreds, we performed volcano plot analyses to compare the proteomes of B73 and EA2024 in control conditions and upon drought (**Figure 2A**). In control conditions (left volcano), we identified 93 over- and 132 under-accumulated proteins in B73 compared to EA2024. Upon drought (right volcano), the number of affected proteins increased, with 250 over- and 189 under-accumulated in B73 with respect to EA2024.

We then used all these DAPs to perform a Venn diagram and determine which of them are differentially abundant in one or both comparisons (**Figure 2B**). We observed that 93 proteins have a different abundance between inbreds under control conditions but not under drought. We defined this behavior as a convergent response. On the other hand, 307 proteins have similar abundances between inbreds in control conditions but differ under drought. We defined this behavior as a divergent response. In addition, a subset of 132 proteins has a different abundance in both control and drought comparisons (**Figures 2B, C**). Among them, 74 have enhanced differences upon drought (32 under- and 42 over-accumulated) and were also considered as having a divergent response, while 7 proteins (3 under- and 4 over-accumulated) have an attenuated difference and were considered as having a convergent response. The remaining 51 proteins (27 under- and 24 over- accumulated) have no changes in the differential abundance in both comparisons, and therefore, they were not considered to be affected by drought. Overall, we identified 481 DAPs affected by drought, 100 (20.8% of the total) of them having a convergent response and 381 (79.2%), a divergent response.

### 3.3 Convergent and divergent responses reveal proteomic reprogramming in B73 under drought

**Figure 3:**
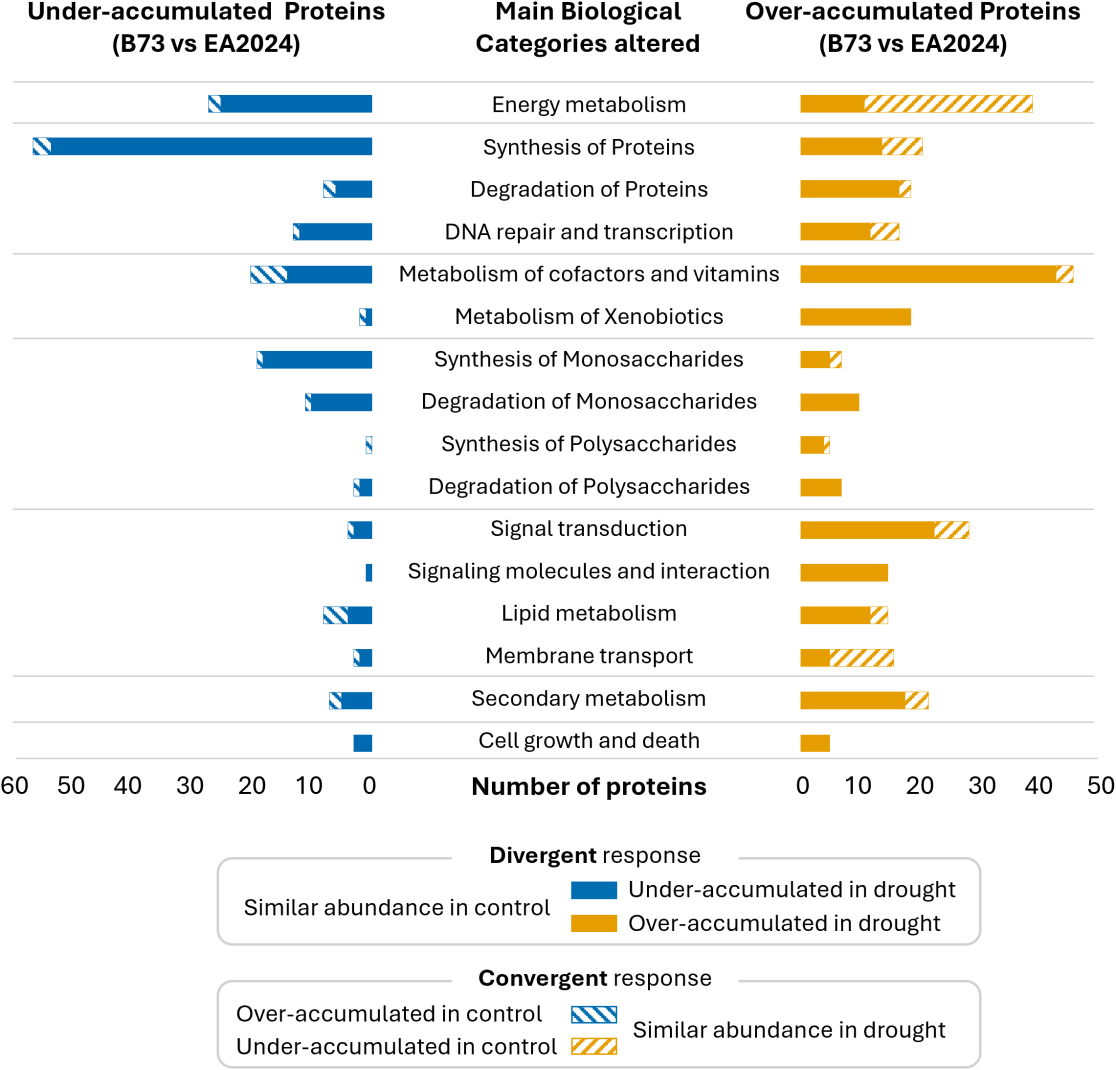
Functional enrichment of proteins showing divergent or convergent responses between B73 and EA2024 under drought conditions. The central panel shows enriched biological categories identified from proteomic comparisons between B73 and EA2024. The divergent and convergent responses are explained at the bottom.

To identify the metabolic pathways associated with the 481 DAPs between B73 and EA2024, we classified them according to functional categories (**Figure 3**, **Supplementary Table 2**). Our results revealed that 289 DAPs (60.1% of total affected), belong to three major biological processes: energy metabolism, gene expression and protein turnover (including protein synthesis and degradation, DNA repair, and transcription) and detoxification and cofactor metabolism. Additional categories present in our DAPs dataset include carbohydrate metabolism, covering the synthesis and degradation of monosaccharides and polysaccharides, as well as signaling and membrane-associated processes, secondary metabolism, and cell growth and death (**Figure 3**).

Out of the 100 convergent DAPs, 73 of them are under-accumulated in B73 with respect to EA2024 under well-watered conditions and increase their abundance in B73 upon drought reaching the EA2024 basal levels (**Figure 3**). The largest number of convergent DAPs belong to the category of energy metabolism and are mainly associated with the upstream elements of the photosynthetic machinery. These proteins are related to the light harvesting processes in the chloroplast, like photosystem I and II (PSI and PSII) components, chlorophyl, and cytochrome B6 components. In addition, another major group of convergent DAPs is related to the membrane transport category, including transporters localized to multiple cellular compartments such as the vacuole, mitochondria, chloroplasts, and plasma membrane (**Supplementary Table 2**). The remaining 27 convergent proteins are, in contrast, over-accumulated in B73 with respect to EA2024 in well-watered conditions and decrease their abundance in B73 upon drought, reaching the EA2024 basal levels. These proteins are similarly distributed across different functional categories (**Supplementary Table 2**).

Out of the 381 divergent DAPs that have similar abundance in both inbreds under well-watered conditions, 159 (41.7%) are under-accumulated in B73 upon drought, and are mainly related to energy metabolism and gene expression and protein turnover. The divergent proteins associated with energy metabolism are mainly involved in downstream processes of the photosynthetic machinery and energy production processes. Among them, we identified proteins related both to components of the photosystems I and II and chlorophylls and to ATP and NADPH synthesis. (**Supplementary Table 2**). In the same line, gene expression and protein turnover also represents one of the most abundant groups of divergent DAPs that are under-accumulated in B73 with respect to EA2024 upon drought. These DAPs mainly belong to small and large ribosomal subunit proteins and elongation factors (**Supplementary Table 2**). A smaller subset of these divergent DAPs belongs to carbohydrate metabolism, which includes proteins involved in the synthesis and degradation of mono- and polysaccharides. On the other hand, the remaining 222 (58.3%) divergent DAPs that are, in contrast, over-accumulated in B73 with respect to EA2024 upon drought mainly belong to the category of metabolism of cofactors and vitamins, including several proteins related to detoxification and redox mediation. In addition, we also identified over-accumulated proteins related to secondary metabolism, specifically lignin-related proteins, and to signaling and membrane-associated processes, mainly involved in hormone signaling.

## 4 Discussion

In this study, we performed a comparative analysis to investigate changes in the proteome of two contrasting stalk-lodging resistance maize inbreds, B73 (resistant) and EA2024 (susceptible) (Manga-Robles et al., 2021), that also have contrasting responses to drought, being B73 sensitive and EA2024 tolerant (Calderone et al., 2024). Even though post-translational modifications modulate enzyme activity under stress without implying changes in protein abundance (Martí-Guillén et al., 2022), our proteomic analysis allowed us to identify different strategies of these two inbreds in response to drought. While EA2024 maintains a stable proteome with minimal remodeling upon drought, B73 proteome is affected (**Figure 1**, **Supplementary Table 1**). This suggests that the tolerant EA2024 inbred has a pre-adapted, stress ready proteomic state, while this stress impacts the metabolic functions of the drought-sensitive B73 inbred. This observed pattern is consistent with previous works comparing tolerant and sensitive maize inbreds, where tolerant lines maintain a higher degree of metabolic stability under drought stress (Li et al., 2021).

Some proteomic alterations observed in B73 are aimed at redirecting part of its metabolic reorganization towards the basal proteomic state of EA2024. We called this the convergent response (**Figure 2**). These proteins, that are mainly under-accumulated in B73 in control conditions, increase their abundance reaching levels similar to those in EA2024 upon drought. Most of these proteins belong to upstream elements of the photosynthetic machinery and membrane transporters (**Figure 3**, **Supplementary Table 2**). It is well known that photosystems are highly drought sensitive, as reductions in CO_2_ availability increase the excitation pressure in the chloroplast, leading to the over-reduction of the electron transport chain and promoting ROS formation. This situation de-stabilizes light harvesting complexes and impairs their repair cycles (Qiao et al., 2024; Hu et al., 2023). In a previous work, we determined the photosynthetic parameters of these two inbreds and observed that, in well-watered conditions, B73 and EA2024 have similar levels of PSII quantum yield and electron transport rate (Calderone et al., 2024). Thus, the observed over-accumulation of several photosynthetic proteins in EA2024 in well-watered conditions is not translated into a higher photochemical activity and suggests a pre-adapted stress-ready state. As EA2024 does not modify its proteome, this inbred experiences a stronger decline of the previously mentioned photochemical parameters than B73 in drought conditions (Calderone et al., 2024). Thus, our results suggest that B73 increases the abundance of upstream photosystem proteins (convergent response), potentially as a reactive reinforcement of the photosynthetic apparatus to mitigate the impact of water deficit. We observed a similar convergent response concerning membrane transporters. These proteins are involved in water homeostasis, osmotic adjustment, and metabolic transport in response to drought (Schulze et al., 2021; Jiang et al., 2019, 2025). Thus, the observed convergent alteration of the membrane transporters in B73 may be indicating an attempt of this inbred to mimic EA2024 and recover osmotic balance and stabilize cellular water contents in response to drought.

In contrast to the convergent response, we observed a set of proteins whose abundance was similar between both inbreds in well-watered conditions but altered only in B73 upon drought. We called this a divergent response (**Figure 2**). This divergent response of B73 could explain why this inbred is sensitive to drought. Most of these divergent proteins are under-accumulated in B73 with respect to EA2024 upon drought. These proteins mainly correspond to downstream components of the energetic machinery, such as ATP and NADPH-related proteins, although it also includes some upstream elements like PSI and PSII components (**Figure 3**, **Supplementary Table 2**). In addition, other divergent under-accumulated proteins in B73 are related to ribosomal subunits and carbohydrate metabolism. The observed decline in ribosomal proteins agrees with previous reports that pinpoint ribosomal downregulation as an indicator of drought sensitivity in plants (Yadav et al., 2024). Thus, although B73 maintains a higher photochemical performance under drought than EA2024 (Calderone et al., 2024), the divergent decrease in ATP/NADPH-related proteins and ribosomal components suggests a limitation in downstream metabolic sinks rather than impairment of the photosystems themselves. Indeed, B73 exhibits lower intercellular CO_2_ concentration than EA2024 upon drought (Calderone et al., 2024). This suggests a limitation in downstream CO_2_ fixation in B73 and a concomitant overreduction of the photosynthetic electron transport chain, potentially enhancing ROS production via the Mehler reaction (Cruz de Carvalho, 2008; Agurla et al., 2018). Accordingly, we observed a divergent over-accumulation of cofactor and antioxidant related proteins in B73. This may reflect a compensatory mechanism of B73 to mitigate redox pressure under drought. Nevertheless, it cannot be excluded other explanations for low intercellular CO_2_ in B73 under drought, such as genotype specific stomatal behavior, which may also influence water transport and gas exchange processes (Benešová et al., 2012).

In addition, drought-induced hormonal signals (particularly ABA and its crosstalk with cytokinins and jasmonates) are known to affect protein synthesis and turnover (Yang et al., 2017; Hai et al., 2020; Riemann et al., 2015). In our proteomic analysis, we observed a divergent over-accumulation of proteins related to signal transduction in B73 under drought, including several hormone responsive proteins. Thus, this suggests that the different hormone-driven stress responses of these inbreds could trigger distinct proteome remodeling, degradation and turnover, and therefore explain their different response to drought.

In sum, we propose that drought tolerance is achieved through a stress-ready proteomic state present in EA2024 but absent in B73. Our findings suggest that such differences may be linked to contrasting agronomic strategies between the two inbred lines, potentially involving trade-offs between drought responsiveness and structural traits such as stalk-lodging resistance. Future analyses including a wide range of maize inbred lines would be necessary to evaluate the existence of a correlation between stalk-lodging resistance and drought sensitivity.

## Data Availability

The proteomics data generated in this article is available in ProteomeXchange and can be accessed with identifier PXD065315.
