# Supplementary material for: Unravelling distinct proteome changes in two maize inbreds with contrasting stalk‐lodging resistance upon drought
Source: Front Plant Sci. 2026 May 5;17:1830066. doi: 10.3389/fpls.2026.1830066 (PMC13183843; doi:10.3389/fpls.2026.1830066)
Supplement: Supplementary file 1 [file DataSheet1.docx]

**
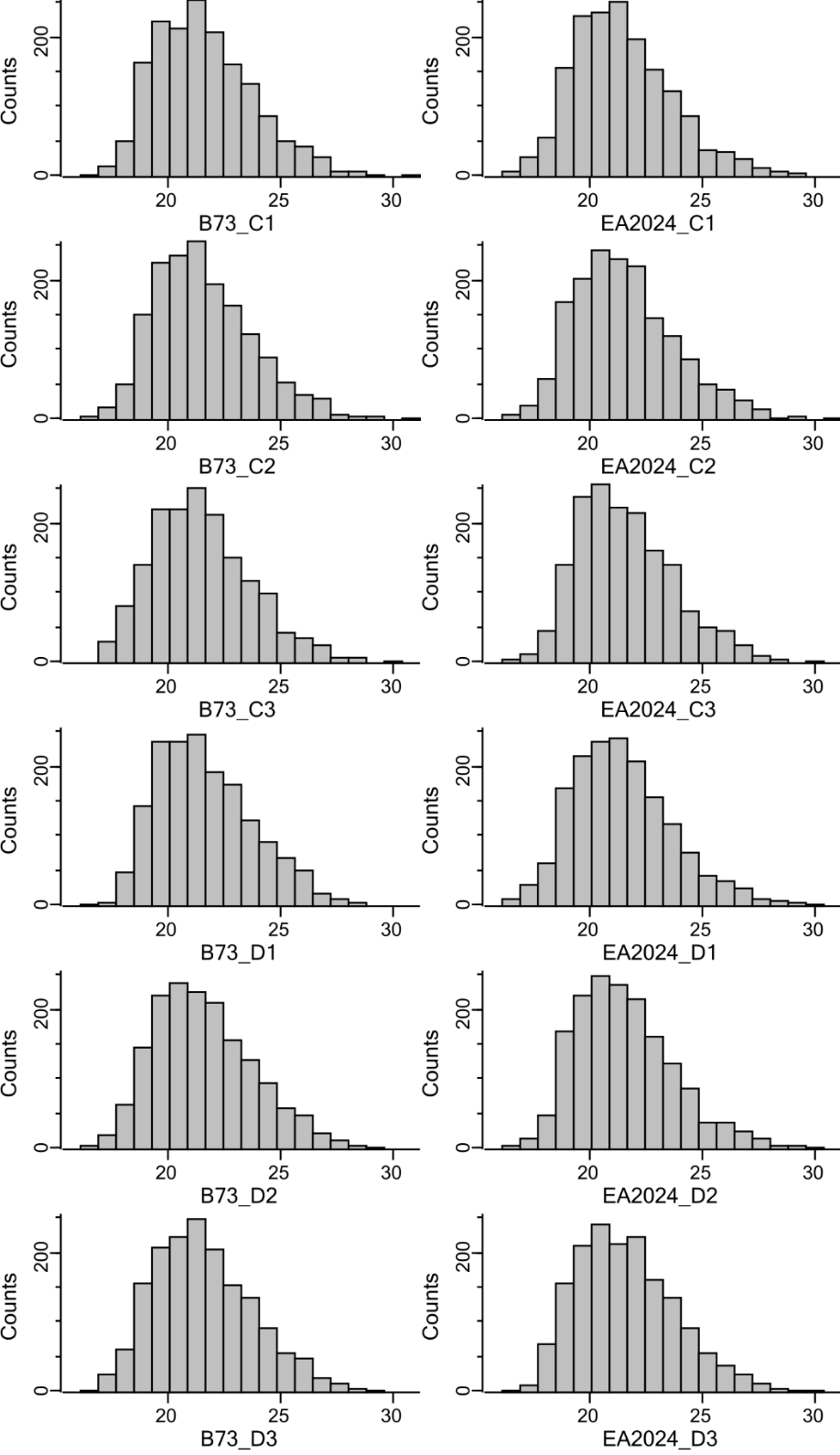
Supplementary Figure 1: Histograms of the distribution of protein abundances.** Histograms of the log protein intensity for each biological replicate used in this study showing normally distributed data. Each graph represents the data distribution of a biological replicate from control (C1, C2 and C3) and drought-stressed plants (D1, D2 and D3).


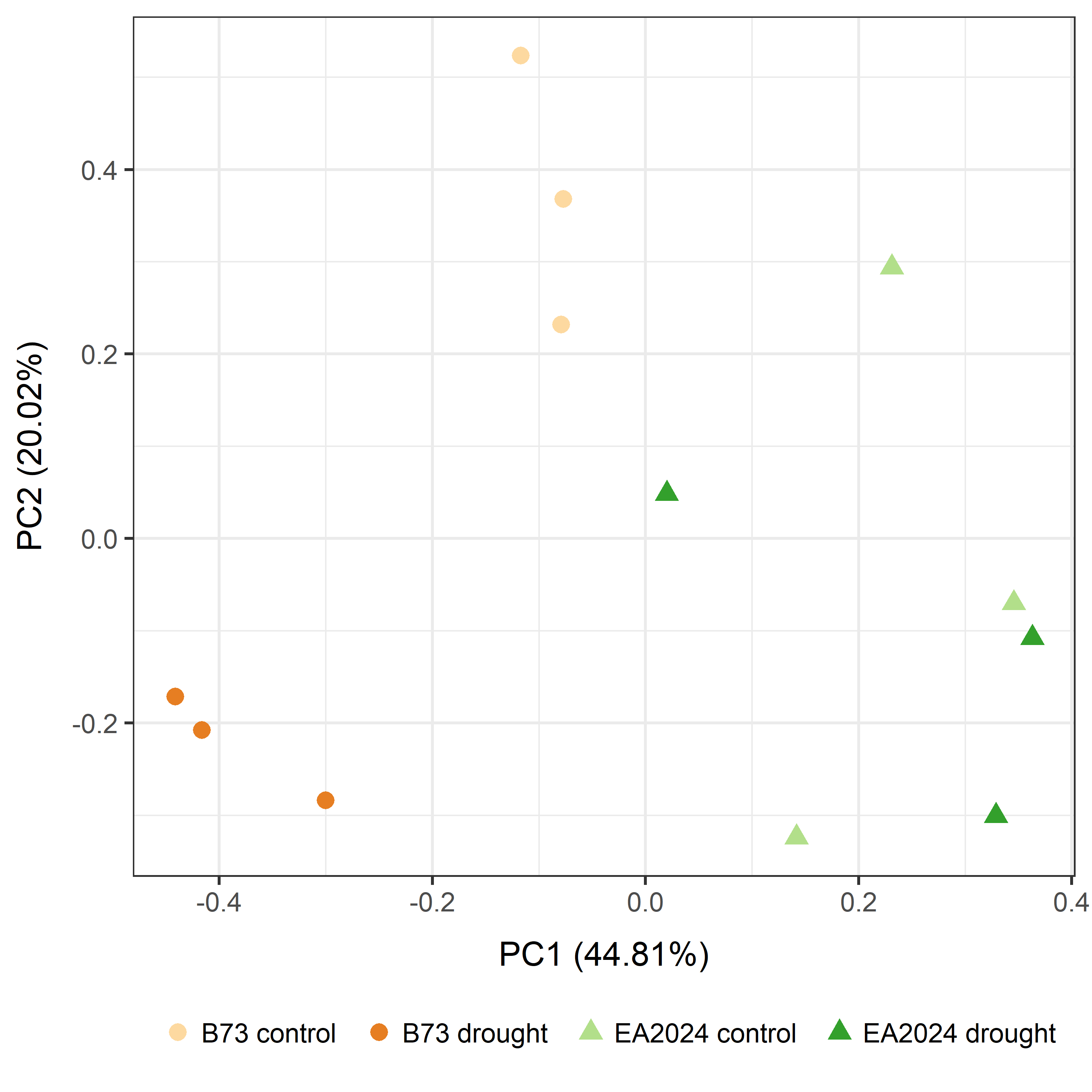


**Supplementary Figure 2. Principal component analysis of protein abundance profiles across all conditions.** Each symbol represents a biological replicate: circles for B73 (light orange = control; dark orange = drought) and triangles for EA2024 (light green = control; dark green = drought).
